# Supplementary material for: Free-living human cells reconfigure their chromosomes in the evolution back to uni-cellularity
Source: eLife. 2017 Dec 18;6:e28070. doi: 10.7554/eLife.28070 (PMC5734875; doi:10.7554/eLife.28070)
Supplement: Supplementary file 1. — The expression of XIST was detected by Real-Time PCR (see note). The △△Ct values were 12 and 11 higher in HeLa and A549 than B-cell, which illustrates nearly 2^10 lower expression of XIST gene in HeLa and A549. The primers of XIST and control gene, used for Real-Time PCR are listed. [file elife-28070-supp1.docx]

***XIST* expression in human B-cell, HeLa and A549.**

The expression of *XIST* was detected by Real-Time PCR (see note). The △△Ct values were 12 and 11 higher in HeLa and A549 than B-cell, which illustrates nearly 2^^10^ lower expression of *XIST* gene in HeLa and A549.

|  | CT value of *XIST* | CT value of GAPDH  (Internal control) | △Ct | △△Ct |
| --- | --- | --- | --- | --- |
| B-cell | 23.16 | 19.96 | 3.21 |  |
| HeLa | 31.14 | 16.21 | 14.94 | 11.73 |
| A549 | 30.18 | 16.11 | 14.07 | 10.86 |

## Note: Total RNA was extracted using TRIzol (Invitrogen), treated with DNaseI (Fermentas), and reverse transcribed using Reverse Transcription System (Promega). Quantitative PCR was performed using Maxima SYBR Green/ROX qPCR Master Mix（Fermentas）in triplicate at 95 °C for 10min, followed by 40 cycles consisting of 95 °C for 15 s, 60°C for 30 s. Primers specific for Gapdh were used to normalize for RNA input. The relative expression of each mRNA was calculated using the ΔCt value between the gene of interest and Gapdh (E = 2−ΔΔCt).

## **The primers of *XIST* and control gene, used for Real-Time PCR**

|  | Forward primer | Reverse primer |
| --- | --- | --- |
| *XIST* | TTCCACTCCCTTTGTATTCCAG | CATTAGGTAACCAGCACCCTCT |
| GAPDH | ACAGCCTCAAGATCATCAGC | ATGGACTGTGGTCATGAGTC |
