# Supplementary material for: Free-living human cells reconfigure their chromosomes in the evolution back to uni-cellularity
Source: eLife. 2017 Dec 18;6:e28070. doi: 10.7554/eLife.28070 (PMC5734875; doi:10.7554/eLife.28070)
Supplement: Supplementary file 2. — Observation and Poission expectation of G3 is shown in Figure 4B, and E7 is shown in Figure 4C. Copy number of other small autosomes is also shown. [file elife-28070-supp2.docx]

Copy number of 13 largest autosomes in 6 HeLa sublines measured by low coverage whole genome sequencing . Observation and Poission expectation of G3 is shown in Fig. 4B, and E7 is shown in Fig.4C . Copy number of other small autosomes is also shown.

| Chromosome | E6 | B8 | E3 | G2 | G3 | E7 |
| --- | --- | --- | --- | --- | --- | --- |
| chr1 | 3 | 3 | 3 | 3 | 3 | 3 |
| chr2 | 3 | 2 | 3 | 2.5 | 2 | 3 |
| chr3 | 2 | 2 | 2 | 2 | 2 | 3 |
| chr4 | 3 | 3 | 2 | 1 | 3 | 3 |
| chr5 | 4 | 4 | 3 | 3 | 3 | 4 |
| chr6 | 2 | 2 | 2 | 2 | 2 | 4 |
| chr7 | 3 | 3 | 3 | 3 | 3 | 3 |
| chr8 | 3 | 3 | 3 | 3 | 3 | 4 |
| chr9 | 3 | 2.5 | 2 | 3 | 3 | 3 |
| chr10 | 3 | 3 | 2 | 2 | 3 | 3 |
| chr11 | 2 | 2.5 | 3 | 2.5 | 3 | 3 |
| chr12 | 3 | 2 | 3 | 3 | 3 | 3 |
| chr13 | 3 | 3 | 3 | 3 | 3 | 3 |
| Average copies | 2.8 | 2.7 | 2.6 | 2.5 | 2.8 | 3.2 |
| λ | 0.8 | 0.7 | 0.6 | 0.5 | 0.8 | 1.2 |

| chr14 | 2 | 2 | 2 | 2 | 2 | 1 |
| --- | --- | --- | --- | --- | --- | --- |
| chr15 | 2 | 2 | 3 | 2 | 2 | 2 |
| chr16 | 2 | 2 | 2 | 2 | 2 | 2 |
| chr17 | 3 | 3 | 3 | 3 | 3 | 3 |
| chr18 | 1 | 2 | 1 | 2.5 | 3 | 3 |
| chr19 | 2 | 2 | 2 | 2 | 2.5 | 2.5 |
| chr20 | 3 | 3 | 3 | 3 | 3 | 3 |
| chr21 | 1 | 1 | 1 | 1 | 1 | 1 |
| chr22 | 2 | 2 | 1 | 2 | 2 | 1 |
