# Supplementary material for: Free-living human cells reconfigure their chromosomes in the evolution back to uni-cellularity
Source: eLife. 2017 Dec 18;6:e28070. doi: 10.7554/eLife.28070 (PMC5734875; doi:10.7554/eLife.28070)
Supplement: Supplementary file 3. [file elife-28070-supp3.docx]

**4-state model that incorporates selection as presented in Fig. 4a.**

The four states are

x_1_ – C(1:2), x_2_ – C(2:2), x_3_ – C(2:3), x_4_ – C(2:4) with the transitions defined below

$x_{1}(T)\begin{matrix} u \\ \rightleftharpoons\\ 2u \end{matrix} x_{2}(T)\begin{matrix} v \\ \rightleftharpoons\\ 3v \end{matrix} x_{3}(T)\begin{matrix} 3v \\ \rightleftharpoons\\ 4v \end{matrix} x_{4}(T)$

$$x_{1}+ x_{2}+x_{3}+x_{4}=1$$

Selective coefficient:

$$\left\{ \begin{aligned} x_{1}:1 \\ x_{2}:1+s \\ x_{3}:1+t \\ x_{4}:1 \end{aligned} \right.$$

Initial condition (**Eq. S1**)：

$$X(0)=(1,0,0,0)$$

Transition and normalization (**Eq. S2**):

$$\frac{dX}{dT}=\left[ \begin{matrix} 1-u & u & 0 & 0 \\ 2u & 1-2u-2v & 2v & 0 \\ 0 & 3v & 1-6v & 3v \\ 0 & 0 & 4v & 1-4v \end{matrix} \right]\times\left[ \begin{matrix} 1 & 0 & 0 & 0 \\ 0 & 1+s & 0 & 0 \\ 0 & 0 & 1+t & 0 \\ 0 & 0 & 0 & 1 \end{matrix} \right]=\left[ \begin{matrix} 1-u & u(1+s) & 0 & 0 \\ 2u & (1-2u-2v)(1+s) & 2v(1+t) & 0 \\ 0 & 3v(1+s) & (1-6v)(1+t) & 3v \\ 0 & 0 & 4v(1+t) & 1-4v \end{matrix} \right]\underset{\Rightarrow}{normalization}\left[ \begin{matrix} \frac{1-u}{1+us} & \frac{u(1+s)}{1+us} & 0 & 0 \\ \frac{2u}{1+s+2vt-2us-2vs} & \frac{(1-2u-2v)(1+s)}{1+s+2vt-2us-2vs} & \frac{2v(1+t)}{1+s+2vt-2us-2vs} & 0 \\ 0 & \frac{3v(1+s)}{1+t+3vs-6vt} & \frac{(1-6v)(1+t)}{1+t+3vs-6vt} & \frac{3v}{1+t+3vs-6vt} \\ 0 & 0 & \frac{4v(1+t)}{1+4vt} & \frac{1-4v}{1+4vt} \end{matrix} \right]$$

The time-dependent solution is

$$X(T)=X(0)\left[ \begin{matrix} \frac{1-u}{1+us} & \frac{u(1+s)}{1+us} & 0 & 0 \\ \frac{2u}{1+s+2vt-2us-2vs} & \frac{(1-2u-2v)(1+s)}{1+s+2vt-2us-2vs} & \frac{2v(1+t)}{1+s+2vt-2us-2vs} & 0 \\ 0 & \frac{3v(1+s)}{1+t+3vs-6vt} & \frac{(1-6v)(1+t)}{1+t+3vs-6vt} & \frac{3v}{1+t+3vs-6vt} \\ 0 & 0 & \frac{4v(1+t)}{1+4vt} & \frac{1-4v}{1+4vt} \end{matrix} \right]^{T}$$

The equilibria are (**Eq. S3**)

$$X\left( T\gg0 \right)=$$

$$[ \frac{12\left( 1+us \right)}{15+6\left( 1+s \right)^{2}+4\left( 1+t \right)^{2}-12\left( u+v \right)s^{2}+24vt\left( s-t \right)} ,$$

$$\frac{6(1+s)(1+s+2vt-2us-2vs)}{15+6{(1+s)}^{2}+4{(1+t)}^{2}-12(u+v)s^{2}+24vt(s-t)} ,$$

$$\frac{4\left( 1+t \right)(1+t+3vs-6vt)}{15+6{(1+s)}^{2}+4{(1+t)}^{2}-12(u+v)s^{2}+24vt(s-t)} ,$$

$$\frac{3(1+4vt)}{15+6{(1+s)}^{2}+4{(1+t)}^{2}-12(u+v)s^{2}+24vt(s-t)} ]$$
